# A successful treatment case of refractory hemorrhagic ulcer with eosinophilic gastritis by endoscopic hand suturing

**DOI:** 10.1002/deo2.207

**Published:** 2023-01-09

**Authors:** Shun Nakagome, Eriko Koizumi, Osamu Goto, Michika Kitamura, Noriyuki Kawami, Kazutoshi Higuchi, Takeshi Onda, Jun Omori, Naohiko Akimoto, Katsuhiko Iwakiri

**Affiliations:** ^1^ Department of Gastroenterology Nippon Medical School, Graduate School of Medicine Tokyo Japan; ^2^ Endoscopy Center Nippon Medical School Hospital Tokyo Japan

**Keywords:** endoscopic hand suturing, endoscopic hemostasis, eosinophilic gastritis, gastric ulcer, ulcer bleeding

## Abstract

A 78‐year‐old man was admitted to our hospital with a tarry stool. Esophagogastroduodenoscopy identified tiny oozing on the greater curvature at the antrum. Despite repeated endoscopic hemostasis by coagulation and clipping, rebleeding occurred. On the third rebleeding, we performed endoscopic hand suturing to completely close the ulcer surface. Biopsy showing massive infiltration of eosinophils at the ulcer edge indicated eosinophilic gastritis. After the endoscopic closure by endoscopic hand suturing, the patient had no symptoms of bleeding thereafter and was discharged 19 days after the procedure by taking oral prednisolone. The patient remained well and was continuously treated with a small dose of steroids in outpatient. This is the first case report of the successful application of endoscopic hand suturing to a refractory hemorrhagic ulcer. Further accumulation of clinical experiences is desired to confirm the usefulness of this technique for the prevention of refractory ulcer bleeding.

## INTRODUCTION

A bleeding ulcer is one of the most common diseases in daily clinical practice. Even though endoscopic hemostasis was performed properly, 5%–10% of cases are likely to rebleed.^1^ Although a complete closure of the ulcer surface is considered effective,^2^ use of conventional clips is insufficient to control bleeding due to the difficulty in closing the ulcer completely and maintaining the closure.

Endoscopic hand suturing (EHS) has been developed as an endoscopic continuous suturing technique by using a barbed suture and a through‐the‐scope type flexible needle holder. In this technique, mucosal layers are continuously sutured in a linear fashion. There have been several reports on the feasibility and efficacy of this technique for iatrogenic mucosal defects after endoscopic submucosal dissection (ESD) so far,^3^, ^4^ however, it has not been applied to hemorrhagic ulcers due to other causes than ESD. Here we report a successful introduction of EHS to refractory hemorrhagic ulcer due to eosinophilic gastritis.

## CASE REPORT

**FIGURE 1:**
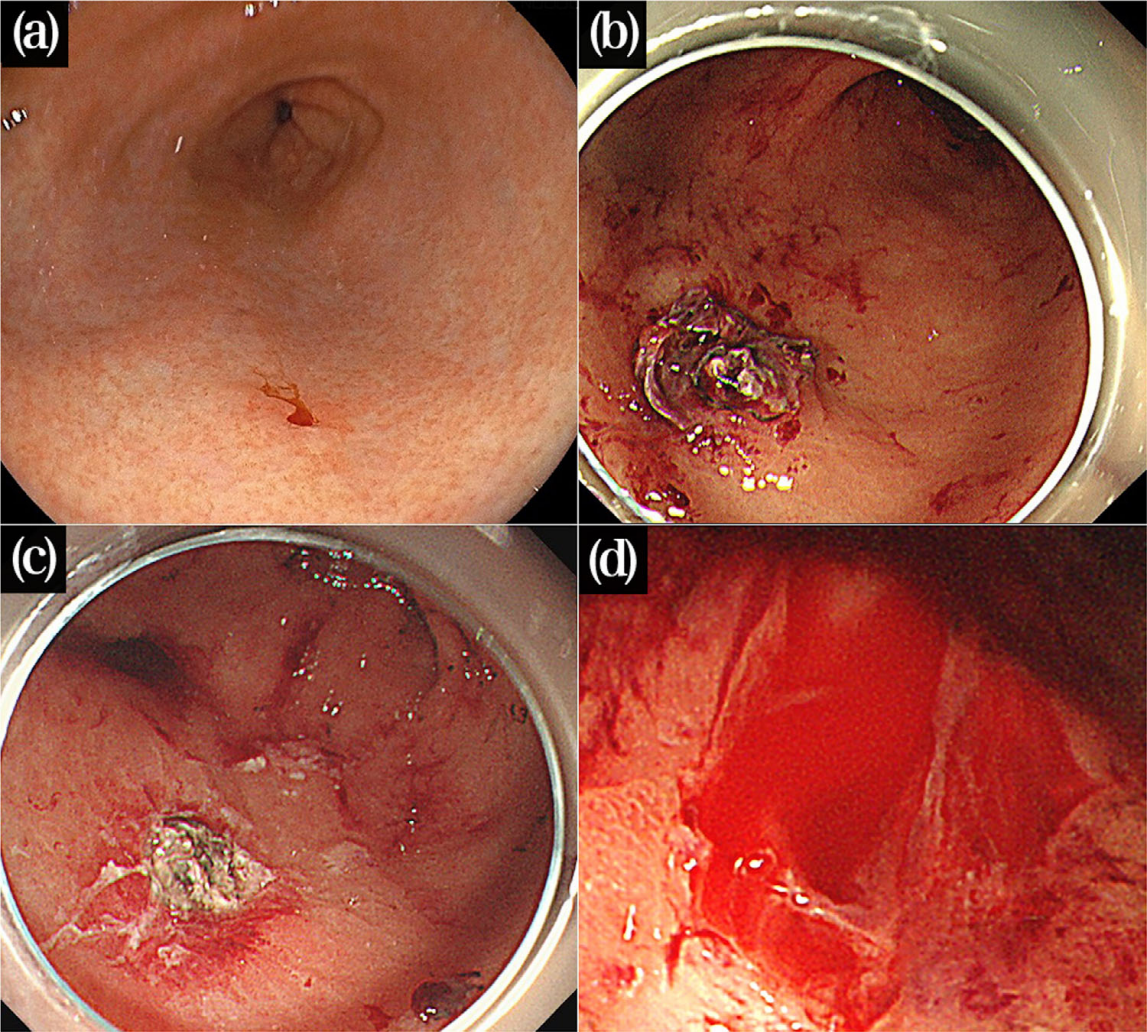
Endoscopic images on bleeding. (a) Initial endoscopy. A tiny oozing was found on the greater curvature of the gastric antrum. (b) The second endoscopy. An exposed vessel was seen at the center of the coagulated site. (c) The third endoscopy. Although the vessel was not visualized clearly, additional coagulation induced pulsative bleeding. (d) The fourth endoscopy just before applying endoscopic hand suturing. Apparent pulsative bleeding was confirmed.

**FIGURE 2:**
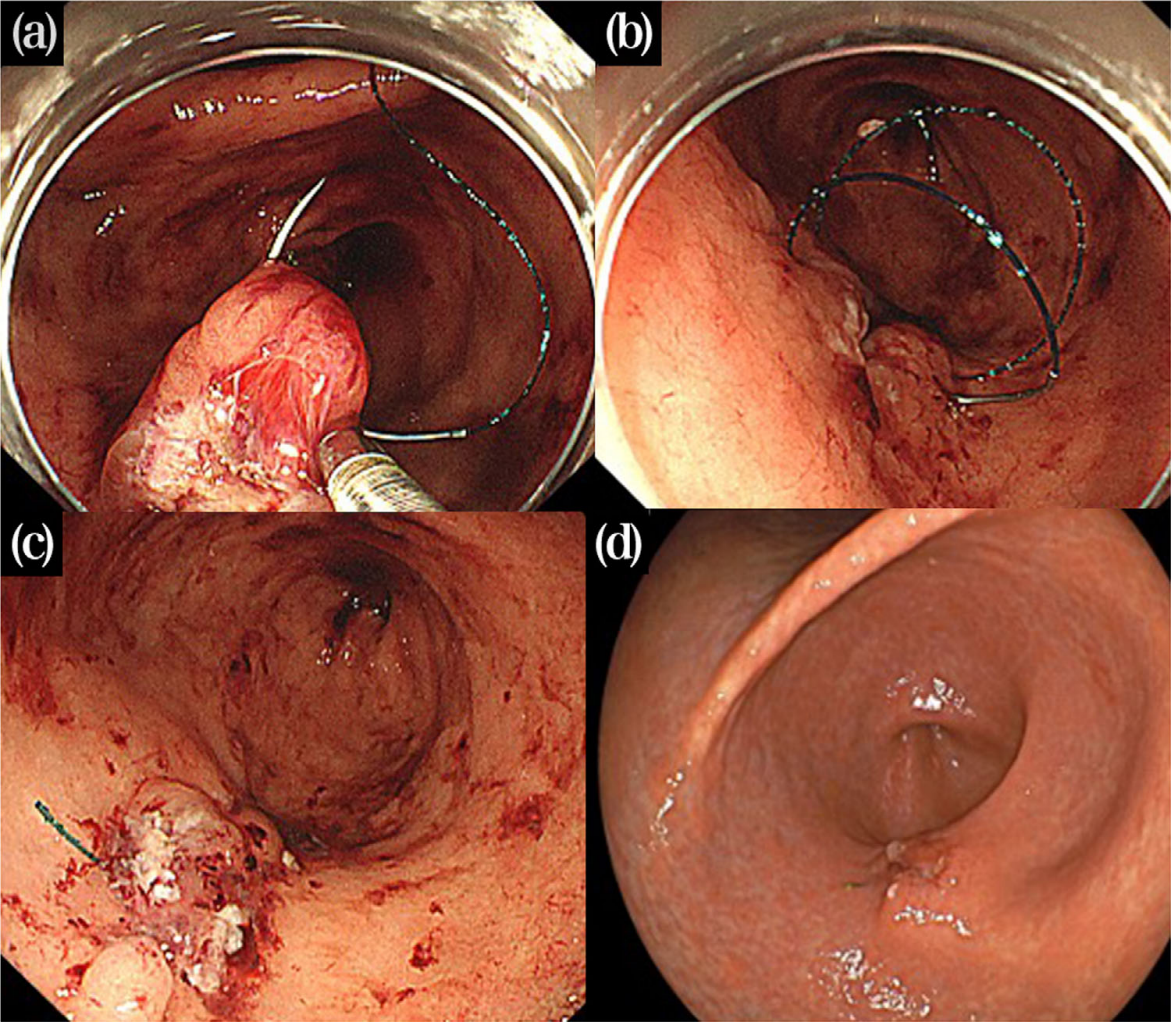
Endoscopic hand suturing applied to a hemorrhagic ulcer. (a) Suturing was initiated at the distal edge of the defect with the needle firmly grasped by the flexible needle holder. (b) After tightening the first suture, continuous suturing was performed in a linear fashion. By pulling the suture, the tissues were tightly apposed. (c) The mucosal defect was completely closed with six stitches in 26 min, and no further bleeding occurred. (d) Follow‐up endoscopy on 3 days after the procedure. The ulcer remained closed.

A 78‐year‐old male was admitted to our hospital with a tarry stool. He had a past medical history of duodenal ulcer, spinal canal stenosis, and lung cancer. He had no regular medication, no recent use of non‐steroidal anti‐inflammatory drugs, and no food allergy. He had a history of eradication therapy for *Helicobacter pylori*. Laboratory tests indicated marked anemia with a hemoglobin level of 7.7 g/dl. White blood cell count was 7000/mm^3^ including a high proportion of eosinophils (11.8%), which was retrospectively noticed (Table 1). Emergency esophagogastroduodenoscopy identified open‐type atrophy and tiny oozing on the greater curvature at the antrum (Figure 1a), and hemostasis was endoscopically obtained by coagulation. After a blood transfusion, intravenous administration of proton‐pump inhibitor was started with no food. However, melena with a decrease in hemoglobin level recurred 8 and 14 days after the admission, respectively, even though endoscopists in charge obtained endoscopic hemostasis in each case, by performing additional coagulation with clipping for an exposed vessel emerged at the center of the previously‐coagulated area (Figure 1b,c). In the third rebleeding on 22 days after the admission (Figure 1d), EHS by using a V‐Loc 180 absorbable barbed suture (VLOCL0604; Covidien, Mansfield, MA, USA) and a flexible needle holder (Olympus, Co., Ltd., Tokyo, Japan) was performed (Video 1). After delivering the suture through the overtube and placing it on the distal edge of the ulcer, the mucosal rim was continuously sutured in a linear fashion. The ulcer was completely closed with 6 stitches in 26 minutes (Figure 2). The remnant suture with the needle was cut with dedicated scissor forceps (Olympus) and retrieved transorally. Thereafter, symptoms of rebleeding were not presented, and the patient resumed having diet four days after the procedure. A *Helicobacter pylori* antibody was negative, and the cause of the ulcer was still unknown. To investigate histological assessments of the ulcer, a biopsy of the ulcer edge was taken at the EHS procedure, which revealed marked infiltration of eosinophilic leukocytes (49/high power field), and finally, we diagnosed it as eosinophilic gastritis (Figure S1). On 28 days after the admission oral steroid administration (30 mg of prednisolone per day) was started. He had a favorable clinical course without rebleeding. After the step‐down administration of steroids, the patient was discharged 41 days after the admission (Figure S2). Biopsy specimens taken on 60 days after the procedure showed a decrease in eosinophilic leukocyte count (4‐5/high power field). The patient remained well under the control of steroid 5 mg per day in outpatient.

**TABLE 1:** Laboratory data on admission

WBC	7,000	/µl	AST	25	IU/L
Neutro	62	%	ALT	25	IU/L
Lympho	27	%	LDH	202	IU/L
Mono	6.8	%	CPK	54	IU/L
Eosino	11.8	%	ALP	65	IU/L
Baso	0.9	%	γ‐GTP	19	IU/L
Hb	7.7	g/dl	T‐Bil	0.37	mg/dl
Plt	31.2	/µl	Na	140	mEq/L
			Cl	108	mEq/L
PT	75.5	%	K	4.3	mEq/L
PT‐INR	1.16		BUN	15.9	mg/dl
APTT	34.3	sec	Cre	0.83	mg/dl
			CRP	0.05	mg/dl
			IgE	94	IU/ml

## DISCUSSION

In this case, we failed to manage ulcer bleeding although appropriate endoscopic treatment was conducted in each procedure before the application of EHS. Compared to conventional hemostatic methods, the following advantages in treating the ulcer by EHS are considered. First, EHS is expected to provide a secure and sustained closure compared to other endoscopic closure techniques based on clipping,^2^ because it is almost identical to surgical suturing, which is considered a basic technique for tissue apposition. Secondly, the ulcer can leave closed until it healed for the reason mentioned above. If the mucosal defect dehisces easily, exposure to various stimuli, such as gastric acid, poses a substantial risk of rebleeding.^3^, ^4^ Therefore, a long‐lasting closure must be helpful for the decrease of rebleeding because of the protection from these stimuli. Indeed, an in‐vivo animal study demonstrated that EHS increased the healing speed of post‐ESD gastric mucosal defects.^3^ In the follow‐up esophagogastroduodenoscopy, the sutured mucosal defect remained closed without dehiscence, therefore protection from various stimuli and preservation of healing condition on the ulcer might bring favorable results in the ulcer healing. Despite these advantages, facilities and procedures that can carry out EHS are still limited. Except for EHS, a purse‐string closure with a detachable snare and clips, or the over‐the‐scope clip closure can be an alternative technique, although they may become difficult to apply when the tissue is fibrotic due to repeated inflammation. In addition, EHS requires a certain level of endoscopic skills. Although there is no evidence of the learning curve on EHS, ESD skills, and sufficient training in ex‐vivo settings may be required for successful EHS.

The precise mechanism of tissue‐damaging eosinophilic gastroenteritis is unknown. In this disease, increased interleukin 5 (IL‐5) by certain stimuli induces generation, differentiation, and activation of eosinophils, and IL‐15/IL‐13 from Th2 cells produces eotaxin‐3 on the epithelium, which promotes infiltration of eosinophils. Eosinophils can release various mediators, such as eosinophilic cationic protein, eosinophilic peroxidase, and major basic proteins which are known to be tissue toxins.^6^ Mast cells, which increase with eosinophilia, may be involved in the pathogenesis of eosinophilic gastroenteritis. Histamine released by mast cell degranulation is a mediator of cellular damage and is one of several factors ascribed to ulcerogenesis.^10^ These multiple factors may let ulcers intractable and resistant to the endoscopic treatments that we performed. It might be thought that frequent endoscopic coagulation prevented the natural healing process of the erosion and accelerated the ulceration. However, we still considered that this refractory bleeding ulcer should be an extraordinary case affected by eosinophil infiltration because primary hemostasis was successfully obtained in all sessions.

In the treatment of eosinophilic gastritis, the systemic oral steroid is generally administered, which is reported to be effective in relieving symptoms. As this patient had no food allergy and the specific cause of eosinophilic gastritis was unknown, we decided to empirically initiate per‐oral steroid treatment. The follow‐up biopsy revealed that the eosinophilic leukocyte count was decreased, which indicates that steroids were effective for the treatment of eosinophilic gastritis and ulcer healing.

It is well known that eosinophilic gastroenteritis is complicated by ulcerative findings, but a bleeding ulcer that is difficult to control is a rare case. Regarding bleeding peptic ulcer with eosinophilic gastroenteritis, six cases were found by searching for “eosinophilic gastroenteritis” and “bleeding ulcer” in Pubmed (Table 2).^5^, ^6^, ^7^, ^8^, ^9^, ^10^ There are more male cases than female cases, and most cases are young. In one case, control of bleeding by clipping was insufficient, then additional interventional radiology was conducted. Steroid administration was also effective in five cases out of six cases.

**TABLE 2:** Previous reports regarding ulcer bleeding due to eosinophilic gastroenteritis

Reference number	First author	Publication year	Age (years)	Sex	Location of ulcer	Hemostasis method	Treatment	Prognosis
^5^	Yamazaki	2015	14	Male	Duodenum	Clipping	Steroid administration	Cure
^6^	Miura	2019	15	Female	Stomach	Clipping	Steroid administration	Cure
^7^	Raithel	2014	22	Male	Stomach	Not performed	Food restriction	Cure
^8^	Priyadarshni	2020	28	Male	Duodenum	Clipping	Interventional radiology	Cure
^9^	Gonzalez‐Canalizo	2019	20	Male	Duodenum	Not performed	Steroid administration	Cure
^10^	Wennmann	2019	79	Male	Stomach	Not performed	Steroid administration	Cure

Unfortunately, the diagnosis of eosinophilic gastroenteritis was delayed due to the delay in taking biopsies: in addition to the delay in noticing abnormal eosinophil numbers in blood tests, the endoscopists hesitated to take biopsies which might induce further bleeding. Moreover, emergency endoscopy was performed by various endoscopists according to a rotating schedule, in which each endoscopist was focusing on managing the bleeding without sufficient understanding of the whole clinical course in this patient. We acknowledged that these issues should be avoided thereafter. Furthermore, a biopsy has been taken from the stomach only at the fourth time of endoscopy. When eosinophilic gastroenteritis was suspected, biopsies from other gastrointestinal tracts should be performed for a more definitive diagnosis.

In conclusion, we experienced a case of eosinophilic gastritis with a refractory hemorrhagic ulcer which was successfully treated by EHS. This is also the first case report that EHS was applied to non‐iatrogenic hemorrhagic ulcers. Accumulation of clinical experience and further investigation is required to confirm the effectiveness of this novel endoscopic technique for persistent ulcer bleeding.

## CONFLICT OF INTEREST

The flexible needle holder and the scissors forceps which were used in this case were complimentarily provided by Olympus Co., Ltd.

## Supporting information


**Figure S1** Histology of biopsy specimen obtained from the ulcer. (a) Histopathological findings (H&E stain, x100). Peptic biopsy findings from the ulcer. Marked eosinophilic leukocyte infiltration in the lamina propria of the stomach was observed. (b) Histopathological findings (H&E stain, x100). Peptic biopsy findings from the ulcer. A decrease in eosinophilic leukocyte infiltration was observed.

**Figure S2** Clinical course during the hospitalization In total, the ulcer bleeding occurred four times, and endoscopic hemostasis was performed respectively. After applying endoscopic hand suturing followed by steroid administration, anemia did not progress and the patient recovered.

**Video 1** Movie of Endoscopic Hand suturing applied to hemorrhagic ulcers In this video, we decided to apply endoscopic hand suturing to the hemorrhagic ulcer 22 days after the admission. First, we performed thermal coagulation on the bleeding ulcer. Then, suturing was initiated at the distal edge of the defect. By continuous suturing, the mucosal defect was completely closed, and the control of bleeding was confirmed.
