# Supplementary figures and images for: A successful treatment case of refractory hemorrhagic ulcer with eosinophilic gastritis by endoscopic hand suturing
Source: DEN Open. 2023 Jan 9;3(1):e207. doi: 10.1002/deo2.207 (PMC9829097; doi:10.1002/deo2.207)

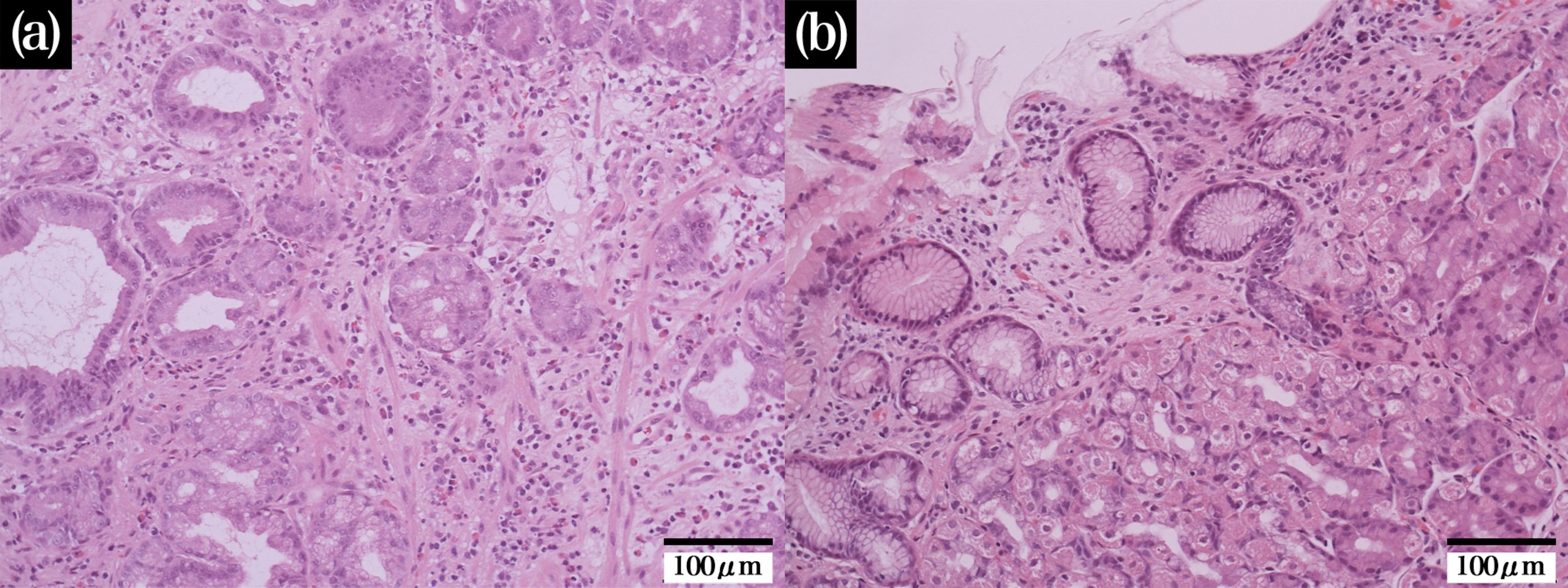

Supplement: Supplementary file 1 — Figure S1 Histology of biopsy specimen obtained from the ulcer. (a) Histopathological findings (H&E stain, x100). Peptic biopsy findings from the ulcer. Marked eosinophilic leukocyte infiltration in the lamina propria of the stomach was observed. (b) Histopathological findings (H&E stain, x100). Peptic biopsy findings from the ulcer. A decrease in eosinophilic leukocyte infiltration was observed. [file DEO2-3-e207-s003.jpg]

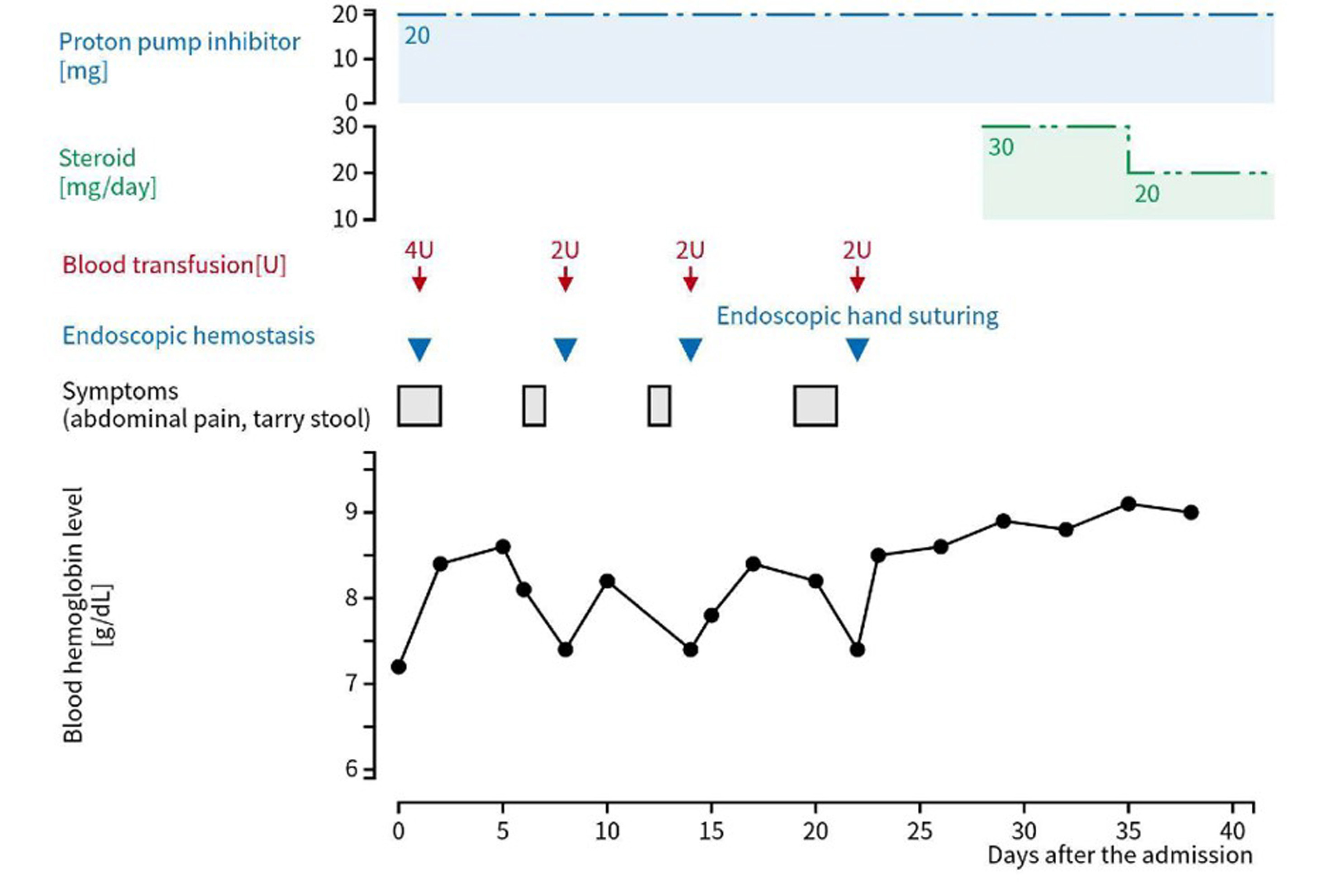

Supplement: Supplementary file 2 — Figure S2 Clinical course during the hospitalization In total, the ulcer bleeding occurred four times, and endoscopic hemostasis was performed respectively. After applying endoscopic hand suturing followed by steroid administration, anemia did not progress and the patient recovered. [file DEO2-3-e207-s002.jpg]
